# Transgenesis of the gonochoristic nematode *Caenorhabditis inopinata* by microparticle bombardment with hygromycin B selection

**DOI:** 10.17912/micropub.biology.000564

**Published:** 2022-05-05

**Authors:** Shun Oomura, Kenji Tsuyama, Nami Haruta, Asako Sugimoto

**Affiliations:** 1 Graduate School of Life Sciences, Tohoku University

## Abstract

The gonochoristic nematode *Caenorhabditis inopinata* is the phylogenetically closest species to the well-studied nematode *Caenorhabditis elegans* (Kanzaki *et al.* , 2018). While *C. inopinata* has been expected to be a useful comparative model for *C. elegans* , efficient transgenesis methods have not been available. Here, we established a method to integrate transgenes into the *C. inopinata* genome by microparticle bombardment with hygromycin B selection. *C. elegans-* derived genetic elements tested in this study, including universal and germline-specific promoters, ORFs, and 3’UTRs, were all functional in *C. inopinata.* Using this method, transgenic *C. inopinata* strains that express fluorescent subcellular markers were established.

**Figure 1. Microparticle bombardment with hygromycin B screening for C. inopinata:**
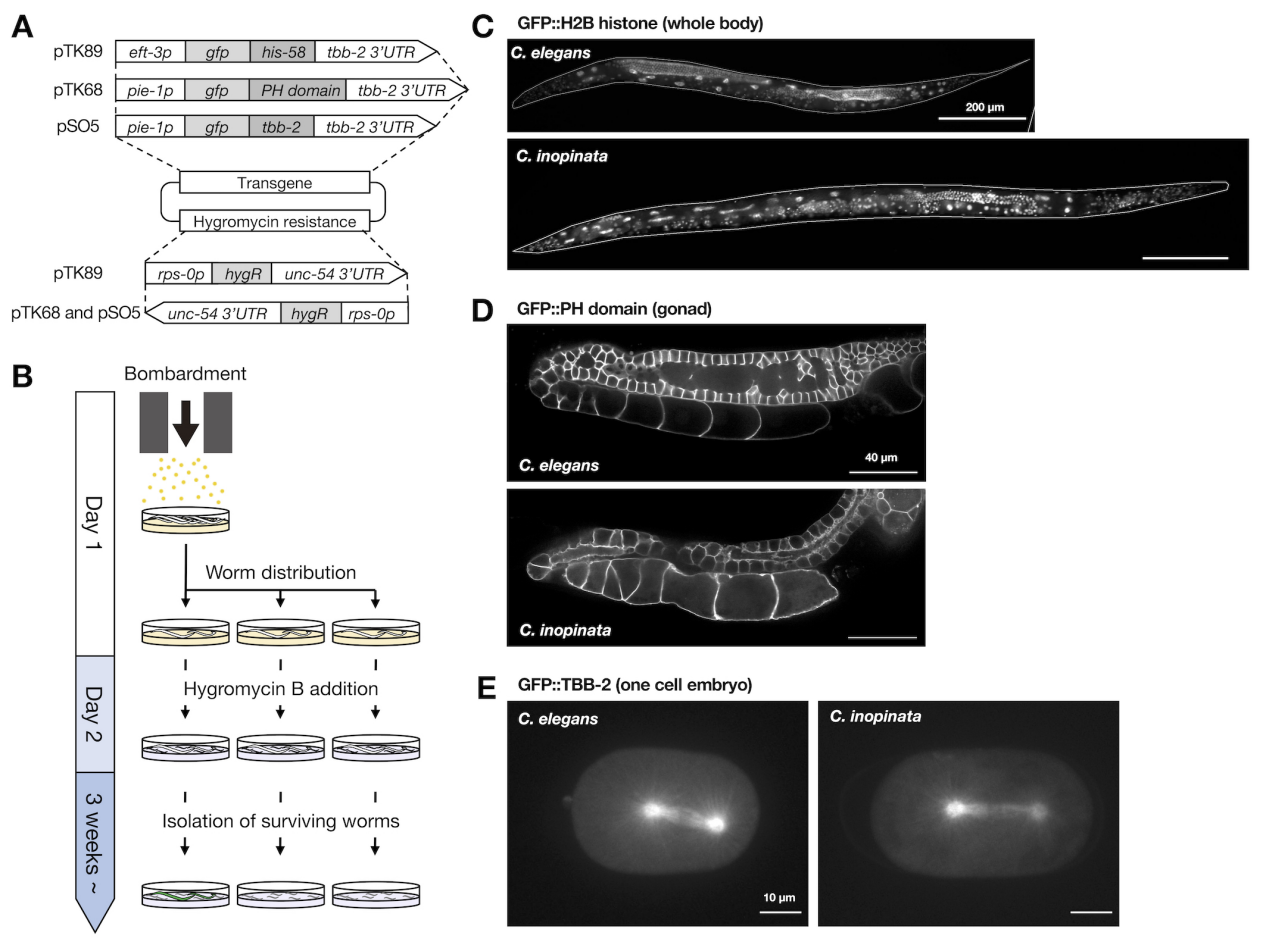
A. Plasmids for microparticle bombardment. They contain transgenes and the hygromycin resistance cassette. B. Time schedule for microparticle bombardment and hygromycin B screening for *C. inopinata* . After the bombardment of young adults of *C. inopinata* , worms were distributed to 10 recovery plates per one shot. On Day 2, hygromycin B was added to each plate. Within three weeks, most non-transformed worms and transient transformants harboring extrachromosomal arrays were killed by hygromycin B, and survivors could be isolated as candidates for transgene integrants. C-E. Fluorescent images of *C. elegans* and *C. inopinata* integrant strains expressing GFP markers. C. GFP::H2B histone in hermaphrodite/female adults. Scale bars = 200 μm. D. GFP::PH domain in adult hermaphrodite/female gonads. Scale bars = 40 μm. E. GFP::TBB-2 in one-cell embryos. Scale bars = 10 μm.

## Description

*C. inopinata* is a recently isolated sister species to *C. elegans* (Kanzaki et al., 2018). Despite their phylogenetic proximity, these two species have various morphological and ecological differences in, for example, body length ( *C. elegans* : 1 mm, *C. inopinata* : 2-3 mm), sex (hermaphroditic vs. gonochoristic), and optimum temperature for growth (20 °C vs. 27 °C). Various microinjection-based transgenesis methods have been developed for *C. elegans* , including the formation of extrachromosomal arrays (Mello et al., 1991), transposon- or CRISPR/Cas9-based gene integration (Frøkjær-Jensen et al., 2008; Dickinson et al., 2013). In these methods, transgene-containing plasmids are injected into adult hermaphrodite gonads. In contrast, microinjection in *C. inopinata* is technically difficult because of the thin and fragile structure of female gonads and the small brood size (average < 20), and transgenesis has been largely unsuccessful. To circumvent this problem, we established a method to integrate transgenes into the *C. inopinata* genome by microparticle bombardment with hygromycin B selection.

Microparticle bombardment using DNA-coated gold particles (microcarrier) has been used to generate low-copy number transgene integrants in various nematodes, including *C. elegans* and closely related *C. remanei* (Praitis et al., 2001; Semple and Lehner, 2014), *Pristionchus pacificus* (Namai and Sugimoto, 2018), and some parasitic nematodes (Jackstadt et al., 1999; Higazi et al., 2002). Combinations of antibiotics and antibiotic resistance genes (e.g., puromycin, G418, hygromycin B) can be applied to screening for integrants (Radman et al., 2013; Semple and Lehner, 2014; Namai and Sugimoto, 2018) *.* To perform the bombardment in *C. inopinata,* we chose hygromycin B and its resistance gene for integrants screening because most worms were effectively killed by 330 μg/ml hygromycin B within 2 to 3 days.

The plasmids for the bombardment were designed to contain a transgene and the hygromycin resistance gene cassette (Fig. 1A). Because of the high level of sequence conservation between *C. inopinata* and *C. elegans* , genetic elements derived from *C. elegans* were applied to express transgenes in *C. inopinata* (Kanzaki et al., 2018). As promoters, the *eft-3* promoter and the *rps-0* promoter (both for universal expression; Hunt-Newbury et al., 2007; Frøkjær-Jensen et al., 2012) and the *pie-1* promoter (for germline-specific expression; Praitis et al., 2001) were used. As universal 3’UTRs, elements from *tbb-2* or *unc-54* were used (Merritt et al., 2008). As for ORFs, *C. elegans* *his-58* (histone H2B), *tbb-2* (β-tubulin) *,* a mammalian PH (Pleckstrin-homology) domain (Audhya et al., 2005), and the GFP sequence modified for *C. elegans* were used.

The protocol for microparticle bombardment and hygromycin B screening for *C. inopinata* was based on the protocols for *C. elegans* (Greiss and Chin, 2011; Radman et al., 2013) and *P. pacificus* (Namai and Sugimoto, 2018) with some modifications (Fig. 1B and Methods). First, the culture conditions were optimized for *C. inopinata.* Worms were grown at 27 °C on the NGM plates containing a higher concentration (2.5%) of agar to avoid burrowing, which were seeded with *Escherichia coli* HT115(DE3). Second, because *C. inopinata* is gonochoristic, a mating process for propagation was added during the hygromycin B screening. If there were <15 survivors per plate on Day 3 after hygromycin B addition, wild-type adults (mixtures of males and females) were added for mating with the survivors. After successful propagation, hygromycin B screening was repeated. If the number of survivors on Day 3 was large enough to mate among them, the plates were left for several weeks to obtain integrants, as in the case of hermaphroditic nematodes *C. elegans* and *P. pacificus* . At least one integrant line was obtained from five to eight shots targeting 2,000 ~ 20,000 worms per shot.

Using this method, we generated three *C. inopinata* strains that express GFP-tagged subcellular markers. In all lines, GFP fluorescence was detected at the expected cellular and subcellular locations, confirming that the genetic elements derived from *C. elegans* were functional in *C. inopinata* . In the GFP::H2B histone strain SA1438 ( *tjIs372* [ *eft-3p::gfp::his-58::tbb-2 3’UTR, rps-0p::HygR::unc-54 3’UTR* ]), the chromosomes and nuclei were visualized in the whole body, including the germline and embryos (Fig. 1C). In the GFP::PH domain strain SA1555 ( *tjIs374* [ *pie-1p::gfp::PH domain::tbb-2 3’UTR, rps-0p::HygR::unc-54 3’UTR* ]), cellular membranes were visualized in the germline of adult females and embryos (Fig. 1D). In the GFP::TBB-2 strain SA1648 ( *tjIs375* [ *pie-1p::gfp::tbb-2::tbb-2 3’UTR, rps-0p::HygR::unc-54 3’UTR* ]), microtubules were visualized in the germline and embryos (Fig. 1E,). While germline silencing of integrated transgenes has been often observed in *C. elegans* and *P. pacificus* (Praitis et al., 2001; Namai and Sugimoto, 2018), all transgene-integrated *C. inopinata* worms in this study stably exhibited GFP fluorescent signals in the germline, implying that gene silencing in *C. inopinata* might be weaker than *C. elegans* or *P. pacificus* .

These results demonstrated that microparticle bombardment with hygromycin B screening is useful for transgenesis in the gonochoristic nematode *C. inopinata* . Since *C. elegans* -derived genetic elements seem generally functional in *C. inopinata* , a large collection of transgene constructs of cell-specific and organelle-specific markers developed for *C. elegans* can be directly utilized to construct *C. inopinata* marker strains. This transgenesis method and marker strains will enable more advanced genetic comparative analysis between *C. elegans* and *C. inopinata* .

## Methods

***C. inopinata* culture**

The wild-type of *C. inopinata* (NKZ35) was cultured at 27 °C on 90 mm plates of modified nematode growth medium (NGM) (50 mM NaCl, 0.25% bacto peptone (gibco), 1 mM CaCl _2_ , 0.5 mM MgSO _4_ , 25 mM pH 6.0 KH _2_ PO _4_ , 5 µl/ml, cholesterol, 20 unit/ml Nystatin in DPBS, 2.5% Bacto agar (BD)) seeded with 1 ml of overnight *Escherichia coli* HT115 (DE3) culture in LB. In this condition, it takes 4 days from an egg to the adult stage.

**Construction of vector plasmids**

Each plasmid for microparticle bombardment contains a transgene and the hygromycin B resistance gene cassette. The pTK89 plasmid for the expression of GFP::H2B histone was constructed by inserting the hygromycin B resistance gene cassette from pDD282 (a gift from Bob Goldstein, Addgene plasmid #66823) (Dickinson et al., 2015) and the transgene ( *eft-3p::gfp::his-58::tbb-2 3’UTR* ) into the pUC19 vector. The pTK68 plasmid for the expression of GFP::PH domain in germline was constructed by inserting the transgene ( *pie-1p::gfp::PH domain::tbb-2 3’UTR* ) into the multi-cloning site (NotI-ApaI) of pCFJ1662 (a gift from Erik Jorgensen, Addgene plasmid #51482). The pSO5 plasmid for the expression of GFP::TBB-2 was constructed by inserting the transgene ( *pie-1p::gfp::tbb-2::tbb-2 3’UTR* ) into the pCFJ910 (a gift from Erik Jorgensen, Addgene plasmid #44481) and by substituting its neomycin resistance cassette with the hygromycin B resistance cassette (from pCFJ1662). Plasmid sequences and maps are available upon request.

**Microscopy**

For the fluorescence imaging, ORCA-R2 Digital CCD camera (Hamamatsu Photonics) on Axioplan 2 imaging microscope (ZEISS) with Zeiss Plan-NEOFLUAR 10x/0.30 objective lens (ZEISS) was used. For the imaging of gonads and embryos, ORCA-R2 Digital CCD camera (Hamamatsu Photonics) on IX71 microscope (Olympus) with UPlanSApo 60x/1.30 silicone oil objective lens (Olympus) and a CSU-X1 spinning disc confocal system (Yokogawa Electric Corporation) was used.

**Protocol for microparticle bombardment and hybromycin B screening**

This microparticle bombardment protocol corresponding to the 5 shots for a single plasmid, is based on the ones developed for other nematode species (Praitis et al., 2001; Greiss and Chin, 2011; Radman et al., 2013; Namai and Sugimoto, 2018) and modified for *C. inopinata* .

**I. Collecting the adult worms for the bombardment**

1. Place the 10 mated *C. inopinata* adult females on 100 NGM 90 mm plates seeded with HT115 and incubate at 27 °C for ~7 days to obtain F2 young adults.

2. ~7 days later, wash out the worms from all plates with 5 ml of M9 buffer (22 mM KH _2_ PO _4_ , 42.3 mM Na _2_ HPO _4_ , 85.6 mM NaCl, 18.7 mM NH _4_ Cl, 1 mM MgSO _4_ ) per plate, and collect them into 50 ml conical tubes.

3. Stand the tubes for 10 minutes at room temperature and let most adult worms settle down.

4. Remove the supernatant containing bacteria, eggs, and larvae using a sterile Pasteur pipette.

5. Collect the precipitated adult worms into a single 50 ml conical tube by decantation.

6. Fill up the tube with M9 buffer.

7. Stand the tube for 10 minutes at room temperature and let most adult worms settle down.

8. Remove the supernatant.

9. Repeat washing steps (6 to 8) at least three times until the buffer appears clear of bacteria, eggs, and small larvae.

10. Transfer the adult worms to a 15 mL conical tube.

11. Centrifuge the tube for 1 minute at 800 × g to precipitate the adult worms and remove the supernatant. At this step, 2-5 ml worm suspension can be obtained. Use 1 ml of the worm suspension (2,000 ~ 20,000 worms) for one shot of bombardment.

12. Keep the worm suspension in the 15 mL conical tube at 22 °C horizontally on the shaker at 100 rpm for aeration until bombardment (The incubation period should not exceed 12 hours).

**II. Washing gold microcarriers**

1. Weigh 35-50 mg of 1.6 μm Gold Microcarriers (Bio-Rad, 165-2264) into a 1.5 ml siliconized tube and vortex.

2. Add 1 ml of 70% ethanol and vortex for 5 minutes.

3. Stand for 15 minutes at room temperature.

4. Centrifuge the tube briefly and remove the supernatant.

5. Add 1ml of distilled water and vortex for 1 minute.

6. Stand for 1 minute at room temperature.

7. Centrifuge the tube briefly and remove the supernatant.

8. Repeat steps 4-6 twice to wash the gold microcarriers.

9. Suspend the gold microcarriers in 500 µl of 50% glycerol and store at 4 °C. (The gold microcarriers in 50% glycerol could be stored for 2 weeks.)

**III. Precipitating DNA onto gold microcarriers**

1. Vortex the gold microcarriers for 5 minutes.

2. Immediately, transfer 400 µl of the gold microcarriers in 50% glycerol to a new siliconized 1.5 ml tube and vortex for 1 min.

3. Add 40 µl of 2 μg/µl DNA, 400 µl of 2.5 M CaCl _2_ , and 160 µl of 0.1 M spermidine.

4. Vortex for 2 minutes.

5. Stand for 1 minute at room temperature.

6. Centrifuge the tube briefly and remove the supernatant.

7. Add 1120 µl of 70% EtOH and gently tap the tube to resuspend the precipitate of the gold microcarriers.

8. Centrifuge the tube briefly and remove the supernatant.

9. Add 1120 µl of 100% EtOH and gently tap the tube to resuspend the precipitate.

10. Centrifuge the tube briefly and remove the supernatant.

11. Add 400 µl of 100% EtOH and gently tap the tube to resuspend the precipitate.

12. Use the gold microcarriers immediately for microparticle bombardment.

**IV. Microparticle bombardment for *C. inopinata***

1. Microparticle bombardment was performed using Biolistic PDS-1000/He Particle Delivery System (Bio-Rad, 165-2257) with Hepta Adaptor (Bio-Rad, 165-2225), 1550 psi Rupture Disks (Bio-Rad, 165-2331), and Hepta Stopping Screens (Bio-Rad, 165-2226).

2. Continue vortexing the gold microcarriers until the end of microparticle bombardment.

3. Wash 35 microcarrier disks (Macrocarriers (Bio-Rad, 165-2335), 7 disks for one shot) with 70% EtOH and 100% EtOH, and dry naturally.

4. Load 6 µl of the gold microcarriers on each of seven microcarrier discs and dry them until it discolors.

5. Soak 1550 psi Rupture Disks (Bio-Rad, 165-2331) in the isopropanol and dry naturally.

6. Spread 1 ml of the worm suspension uniformly onto a well-dried and -cooled NGM 90 mm plate.

7. Carry out the microparticle bombardment according to the manufacturer’s instructions with evacuating the bombardment chamber to 27 In. of Hg.

**V. Recovery after microparticle bombardment**

1. Add 10 ml of M9 buffer onto each bombarded plate and suspend the worms.

2. Dispense 10 ml of the worm suspension onto 10 dried HT115 seeded NGM 90 mm using a siliconized tip.

3. Incubate the plates at 27 °C for one day so that the bombarded worms will mate for propagation.

**VI. Hygromycin B screening**

1. One day after microparticle bombardment, add 10 mg/ml hygromycin B (Invitrogen) onto the plates to a final concentration of 330 μg/ml and dry the plates.

2. Dissolve one unit of OP50 V.2 powder (LabTIE) in 25 ml S medium (100 mM NaCl, 5.74 mM K _2_ HPO _4,_ 44.09 mM KH _2_ PO _4_ , 0.055 mM disodium EDTA, 0.025 mM FeSO _4_ ・7 H _2_ O, 0.010 mM MnCl _2_ ・4 H _2_ O, 0.010 mM ZnSO _4_ ・7 H _2_ O, 0.001 mM CuSO _4_ ・5 H _2_ O, 10 mM Potassium citrate pH 6.0, 3 mM CaCl _2_ , 3 mM MgSO _4_ , 5 mg/l cholesterol).

3. Add the 1 ml of OP50 suspension onto each plate and dry the plates.

4. Incubate the plates at 27 °C.

5. Four days after the bombardment, screen for survivors.

**a) When the number of survivors per plate is <15**

6. Add 5 ml of M9 buffer to each plate and let the surviving worms swim and float. Transfer the surviving worms to a new HT115-seeded NGM 90 mm plate using a sterile Pasteur pipette.

7. Incubate the plates at 27 °C for two days to let the worms grow into the adult stage.

8. Check the transgene expression in the adult worms with a fluorescent dissecting microscope.

9. Isolate the fluorescent-positive worms as integrant candidates.

10. Add wild-type adults (both females and males) onto each plate for mating (female: at least 5 worms, male: five times as many as females)

11. Incubate at 27 °C for propagation.

12. After propagation, repeat hygromycin B selection for confirmation.

**b) When the number of survivors per plate is ≥15**

6. Keep incubating the plates at 27 °C.

7. ~three weeks later, isolate the surviving worms as integrant candidates and check the transgene expression with a fluorescent microscope.

## Reagents

**Table:** 

***C.elegans* strains **		
**Strain**	**Genotype**	**Available from**
SA1161	*tjSi193[eft-3p::gfp::his-58::tbb-2 3’UTR; rps-27p::NeoR::unc-54 3’UTR]*	This work
SA1393	*tjSi208[pie-1p::gfp::PH domain::tbb-2 3’UTR; rps-0p::HygR::unc-54 3’UTR]*	This work
SA250	*tjIs54[pie-1p::gfp::tbb-2; pie-1p::2xmCherry::tbg-1; unc-119+]; tjIs57[pie-1p::mCherry::H2B (his-48); unc-119+]*	CGC (Toya *et al* . 2010)

**Table:** 

***C. inopinata* strains **		
**Strain**	**Genotype**	**Available from**
NKZ35	Wild-type	CGC
SA1438	*tjIs372[eft-3p::gfp::his-58::tbb-2 3’UTR; rps-0p::HygR::unc-54 3’UTR]*	This work
SA1555	*tjIs374[pie-1p::gfp::PH domain::tbb-2 3’UTR; rps-0p::HygR::unc-54 3’UTR]*	This work
SA1648	*tjIs375[pie-1p::gfp::tbb-2::tbb-2 3’UTR; rps-0p::HygR::unc-54 3’UTR]*	This work

**Table:** 

**Plasmids for microparticle bombardment**		
**Plasmid**	**Genotype**	**Description**
pTK89	*eft-3p::gfp::his-58::tbb-2 3’UTR; rps-0p::HygR::unc-54 3’UTR*	Promoters, ORF, and 3’UTR derived from *C. elegans*
pTK68	*pie-1p::gfp::PH domain::tbb-2 3’UTR; rps-0p::HygR::unc-54 3’UTR*	Promoters and 3’UTR derived from *C. elegans*
pSO5	*pie-1p::gfp::tbb-2::tbb-2 3’UTR;rps-0p::HygR:: unc-54 3’UTR*	Promoters, ORF, and 3’UTR derived from *C. elegans*
